# Value of [^18^F]FDG-PET/CT and CA125, serum levels and kinetic parameters, in early detection of ovarian cancer recurrence: Influence of histological subtypes and tumor stages

**DOI:** 10.1097/MD.0000000000010098

**Published:** 2018-04-27

**Authors:** Azahara Palomar Muñoz, José Manuel Cordero García, Mª del Prado Talavera Rubio, Ana Mª García Vicente, Francisco José Pena Pardo, Germán Andrés Jiménez Londoño, Ángel Soriano Castrejón, Enrique Aranda Aguilar

**Affiliations:** a Nuclear Medicine-PET IDI Department, Hospital Universitario de Bellvitge, L’Hospitalet de Llobregat (Barcelona); b Nuclear Medicine Department, Hospital General Universitario de Ciudad Real, Ciudad Real; c Medical Oncology Department, Hospital Universitario Reina Sofía, Córdoba, Spain.

**Keywords:** [^18^F]FDG-PET/CT, CA125, kinetic parameters, ovarian cancer recurrence

## Abstract

To assess the diagnostic accuracy of CA125, its kinetic values and positron emission tomography/computed tomography with 2-deoxy-2-[^18^F]fluoro-d-glucose ([^18^F]FDG-PET/CT), in relation with tumor characteristics for suspected recurrence of ovarian cancer. To evaluate the performance of CA125-related parameters as a selection criteria to perform a [^18^F]FDG-PET/CT.

A retrospective analysis of 69 [^18^F]FDG-PET/CT for suspected recurrence of ovarian cancer was performed. All patients had 2 measurements of CA125, before PET/CT, to calculate kinetic values, as CA125vel (CA125vel = [CA125a − CA125b]/time) and CA125dt (CA125dt = [log_2_ × time]/[logCA125a − CA125b]). Maximum standard uptake value (SUVmax) was calculated. The diagnostic accuracy was calculated for all the variables and the optimal cut-off value of each of them by the receiver-operating characteristics (ROC) analysis. All the tests were compared with tumor characteristics and clinical-radiological evolution during follow-up of at least 6 months.

Fifty-five cases were diagnosed of recurrence (11 with CA125 <35 U/mL), while 14 showed no disease (11 with CA125 < 35 U/mL). All of them were correctly cataloged by PET/CT. CA125, CA125vel, and SUVmax showed higher levels in recurrent patients (mean 129.54 U/mL, 24.58 U/mL per mo, and 8.69 g/mL, respectively) than in nonrecurrent (mean 20.35 U/mL, 0.60 U/mL per mo, and 0.64 g/mL, respectively). No statistical differences in CA125dt were found. Patients with recurrence of high-grade serous carcinoma (HGSC) showed higher CA125 and CA125vel, without differences in the rest of subtypes and International Federation of Gynecology and Obstetrics stages. The ROC analyses for CA125, CA125vel, and CA125dt showed an area under the curve (AUC) of 0.873 (95% confidence interval [CI] 0.77–0.969), 0.903 (95% CI 0.813–0.994), and 0.727 (95% CI 0.542–0.913), respectively, with an optimal cut-off point of 23.95 U/mL, 4.49 U/mL per mo, and 3.36 months, respectively, while for the SUVmax the AUC was of 0.982 (95% CI 0.948–1.000), and the cut-off point of 2. Multivariate regression analysis identified CA125 and CA125vel as predictors of recurrence.

[^18^F]FDG-PET/CT is more accurate than the parameters obtained from the CA125 to detect early recurrence. CA125vel is the most suitable parameter, mainly in HGSC. Levels of CA125vel ≥ 4.49 U/mL per mo facilitate earlier detection by the execution of a [^18^F]FDG-PET/CT. The calculation of these parameters is independent of tumor stage at diagnosis.

## 1 Introduction

Epithelial ovarian cancer (EOC) is a heterogeneous disease. Nowadays, it is widely recognized that the histological subtypes constitute 5 different diseases: low-grade serous carcinoma (LGSC), high-grade serous carcinoma (HGSC), endometrioid carcinoma (EC), mucinous carcinoma (MC), and clear cell carcinoma (CCC). EOC continues being the main cause of death from gynecologic malignancies, although the 5-year survival rate has increased in last decade.^[1]^ The poor survival rate is attributed to frequent persistence or recurrence of disease. However, recurrence varies from 10% for stage I to 85% for stage IV with suboptimal debulking.^[2]^

The cancer antigen 125 (CA125) has an established role as a serum tumor marker, and it has been routinely used for monitoring ovarian cancer patients. Regular measurements during follow-up can detect recurrence of cancer months before symptoms or alterations in the conventional imaging techniques.^[3,4]^ However, CA125 originates from the mesothelial cells of the coelomic epithelium, so it rises in related pathologies, leading to poor sensitivity and specificity of a single determination. Therefore, an elevated CA125 is not always indicative of recurrence and there is no evidence of a survival benefit with early treatment of relapse based on a raised CA125 concentration alone.^[5]^ This forces to discriminate between relapse of ovarian cancer and benign increases. On contrary, a rise of CA125 could mean recurrence, even in cases of serum levels below the range of normality, so that doubling in CA125 from the upper limit of normal means objective progression.^[4]^ In fact, the pattern of serum CA125 level ascent, gradual or abrupt, in patients in complete remission has prognostic value.^[5]^ This suggest that if changes in CA125 are assessed in isolation, but considering variations that occur over the time, recurrence could be detected earlier.^[6]^

Based on the fact that positron emission tomography/computed tomography with 2-deoxy-2-[^18^F]fluoro-d-glucose ([^18^F]FDG-PET/CT) could detect ovarian recurrences, even with low titles of CA125,^[7–9]^ the aim of our work was to assess the accuracy of both techniques, as well as CA125 kinetic values, expressed as CA125 velocity (CA125vel) and CA125 doubling time (CA125dt), and their relation with tumor characteristics at the time of diagnosis. And also, to select the best time to perform a [^18^F]FDG-PET/CT that allows an early detection of recurrent ovarian cancer.

## 2 Materials and methods

We retrospectively enrolled consecutive patients with suspicion of EOC recurrence, during the period of 2007 to 2015, referred to our center in order to perform a [^18^F]FDG-PET/CT. This study was performed with institutional review board approval (Ethical Committee for Clinical Research, Hospital General Universitario de Ciudad Real), and informed written consent was obtained from each patient. All patients had been treated for ovarian cancer according to the extension of the disease, achieving a complete remission after the treatment for at least 6 months. Patients were classified by the histological subtypes of EOC at diagnosis, HGSC, LGSC, EC, MC, and CCC. Likewise, the International Federation of Gynecology and Obstetrics (FIGO) stage at diagnosis was considered^[10]^ and was divided into early or advanced stages that involve stages I and II, and III and IV, respectively.

The suspicion of recurrence was based on elevation of CA125 serum levels, alterations in the conventional imaging techniques (CT or magnetic resonance imaging) and/or by the appearance of symptoms. Inclusion criteria also include CA125 elevation at diagnosis of disease and measurement of CA125 in 2 consecutive occasions, to calculate the kinetic values. Patients referred twice to our center, with a time difference exceeding 6 months, that meet the inclusion criteria were included in the analysis in both cases. Platinum-resistant patients (the tumor recurs <6 months after completing chemotherapy) or platinum-refractory (the tumor progresses during the adjuvant chemotherapy), and patients referred to assess response to treatment or suspected progression, were excluded.

The kinetic values of CA125 were evaluated, using 2 consecutive measurements. CA125vel was calculated by the following formula: (CA125a − CA125b)/Time, where CA125a is the CA125 serum level closest to the PET/CT scan, measured at most 1 month around, CA125b is the immediately previous to CA125a, and Time is the time between these 2 measurements, expressed in months. CA125dt was calculated by natural log of 2 (0.693) divided by the slope of the relationship between the log of CA125 and time of CA125 measurement for each patient. The CA125 cut-off value for recurrence is fixed at 35 U/mL.

A hybrid PET/CT scanner was used to obtain images 60 min after administration of 370 MBq of [^18^F]FDG, from the orbital-metal line to the proximal third of the lower limbs. First, low-dose CT images were acquired (120 kV, 80 mA), followed by PET in 3D mode with an acquisition time of 3 min per bed. The [^18^F]FDG-PET/CT images were revised by 2 experienced nuclear medicine physicians, unaware of the clinical data, and the diagnosis was reached by consensus. They were classified as normal if there was no uptake outside expected physiological locations. All the areas presenting an increased tracer uptake above the background, not concordant in the CT images with areas of physiological activity or changes suggestive of infectious or inflammatory processes, were considered positive, and therefore compatible with relapse. The lesion with higher activity was measured by maximum standard uptake value (SUVmax). In those cases of normal [^18^F]FDG-PET/CT, SUVmax was considered 0.

The final diagnosis of recurrence was made by histological confirmation or clinical-radiological follow-up longer than 6 months, further increase in tumor marker levels without treatment, clinical worsening of the patients’ condition, or a response to therapy in the conventional imaging techniques. Instead, nonrecurrence cases were finally cataloged if there was no progression within 6 months in absence of treatment or if histological analysis confirmed the benign nature of the lesions.

Statistical analysis of data was carried out using IBM SPSS Statistics v. 19.0 (IBM Corp., Armonk, NY). Normally distributed continuous variables were compared using *t* test for independent samples. The analysis of variance test was used for defining the difference among subsets of patients, in relation to histological subtypes and stages, early or advanced, at diagnosis. The receiver-operating characteristics (ROC) curve was generated and assessed to find the best cut-off point for CA125, CA125vel, and CA125dt to predict recurrence detected by [^18^F]FDG-PET/CT. A multivariate analysis was used to identify variables associated with recurrent disease. Values of *P* < .05 were considered statistically significant.

## 3 Results

During the studied period, we performed 69 [^18^F]FDG-PET/CT scans in 58 patients [mean (standard deviation [SD]) age 59.96 (12.6) years] with suspicion of recurrent EOC, who meet the inclusion criteria. Patients and tumor characteristics are shown in Table 1.

**Table 1:**
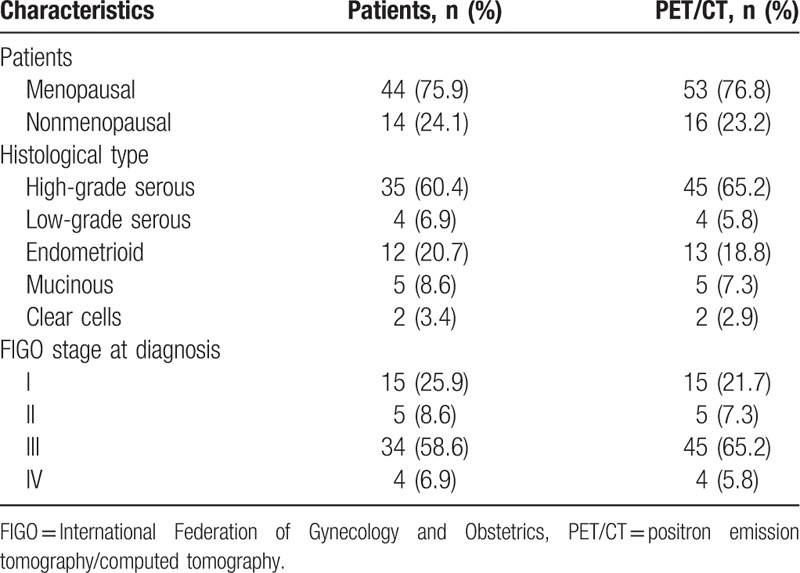
Clinical characteristics.

From these, 55 studies of 45 patients showed pathological [^18^F]FDG uptake and were finally diagnosed of ovarian cancer recurrence. Among them, in 20 patients (5 of them twice), recurrence was verified after surgery or histological analysis, while the remaining 30 cases were diagnosed by clinical and radiological follow-up (mean 28.42 months, SD 19.50). In 14 cases, a normal distribution of [^18^F]FDG was observed, categorizing them as nonrecurrence, which was confirmed in the clinical and radiological follow-up. All cases histologically confirmed showed tumor disease, without false negative results. Although in 9 cases, considered as true positive, there was a combination of tumor and inflammatory pathology. At follow-up, all patients with active treatment presented progression (9 cases) or response to chemotherapy administered (21 cases). On the other hand, in the follow-up of untreated patients, no disease was observed. Therefore, considering the analysis by patients that entails a sensitivity and specificity of 100% for PET/CT.

Among the cases with CA125 serum level below the normal limit (35 U/mL), 11 out of 22 (50%) were finally diagnosed with recurrence, while from the 47 cases of CA125 > 35 U/mL recurrence was detected in 44. This results in a sensitivity of 80% (66.63–89.13%), a specificity of 78.57% (48.82–94.29%), and an accuracy of 79.71% (67.98–88.08%). Table 2 shows the diagnostic accuracy for both tests.

**Table 2:**
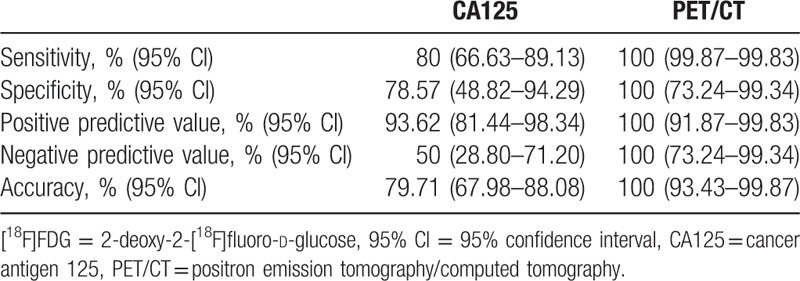
Diagnostic accuracy per patient of CA125 serum levels and visual [^18^F]FDG-PET/CT for recurrence ovarian cancer.

The recurrent group showed higher levels than the nonrecurrent 1 for SUVmax (mean 8.69, SD 3.64 vs. 0.64, SD 1.67, *P* < .001), CA125 (mean 129.54, SD 169.48 vs. 20.5, SD 22.14, *P* < .001), and CA125vel (mean 24.58, SD 45.40 vs. 0.60, SD 3.34, *P* < .001), while the CA125dt had not shown statistical differences (mean 2.16, SD 27.41 vs. −17.25, SD 43.67, *P* = .13).

**Figure 1:**
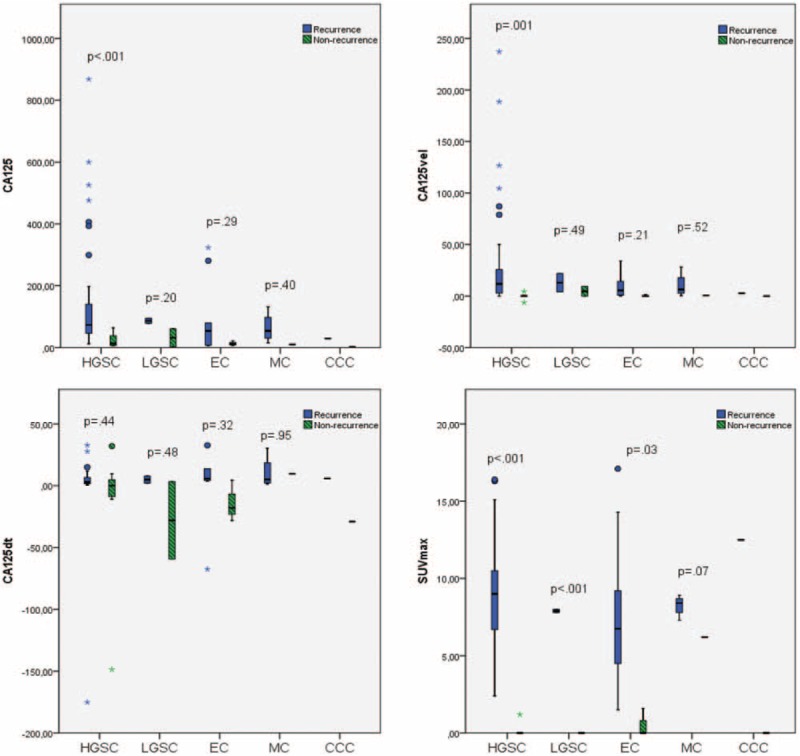
Mean of cancer antigen 125 (CA125), kinetic values of CA125 (CA125vel and CA125dt), and maximum standard uptake value (SUVmax) regarding the histological subtypes, in recurrence and in nonrecurrence cases.

There was not statistical significance in the relation between the histological subtypes, attending to CA125 (*P* = .49), its kinetic values (CA125vel, *P* = .49; CA125dt, *P* = .87), or SUVmax (*P* = .53). We subdivided each histological subtype in recurrence and nonrecurrence group (Fig. 1). There were no differences in relation to the CA125dt. Statistical significance was only observed in the HGSC for the CA125 serum level, CA125vel, and SUVmax, and in the LGSC and the EC for the SUVmax.

**Figure 2:**
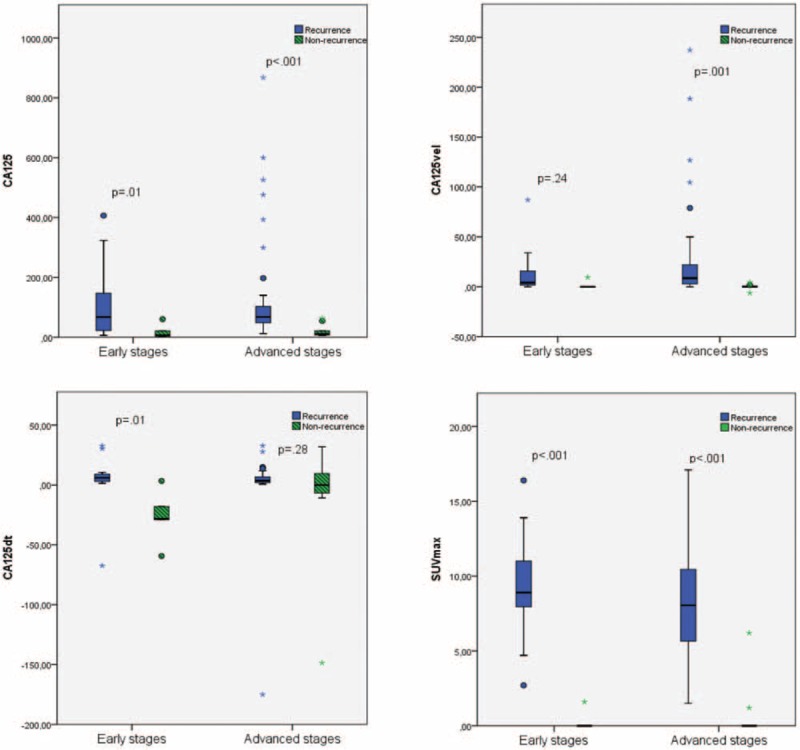
Comparison of the cancer antigen 125 (CA125), kinetic values of CA125 (CA125vel and CA125dt), and maximum standard uptake value (SUVmax) in different tumor stages (early [n = 20] and advance [n = 49]) at the time of diagnosis.

In relation to the stage at diagnosis, early or advanced, there were no statistical differences between levels of CA125 (*P* = .54), CA125vel (*P* = .27), CA125dt (*P* = .77), and SUVmax (*P* = .96). However, when we compared recurrent and nonrecurrent disease, we found significant differences in the early stages in SUVmax, CA125, and CA125dt, and in the advanced stages in SUVmax, CA125, and CA125vel. There were no differences neither in the CA125vel in early and the advanced stages nor in the CA125dt. These results can be seen in Fig. 2.

**Figure 3:**
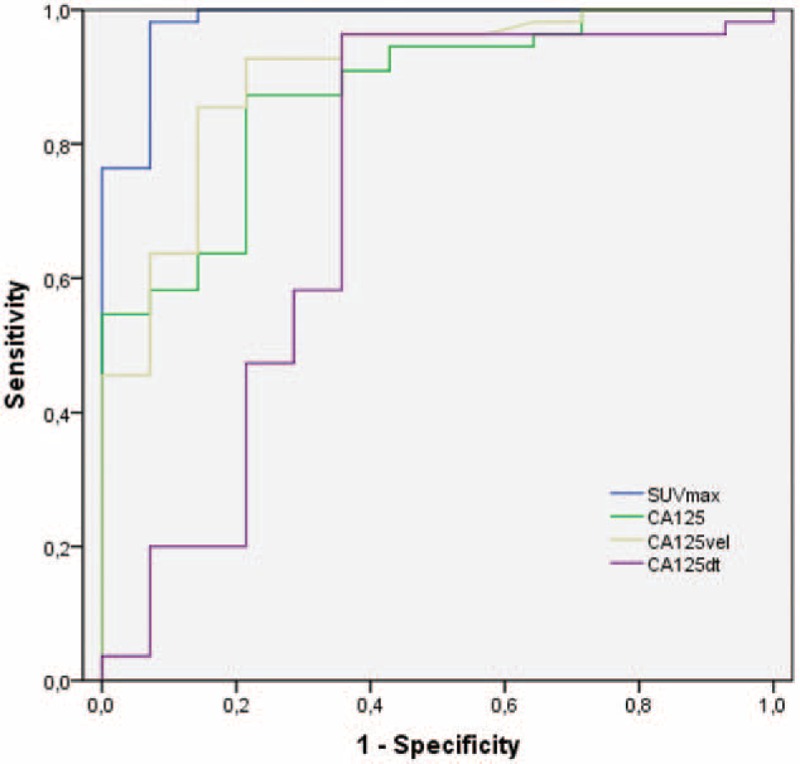
The ROC analysis demonstrated an optimal cut-off value for cancer antigen 125 (CA125) serum level of 23.95 U/mL, for the CA125dt of 3.365 months and for the CA125vel of 4.49 U/mL per mo. The AUCs for CA125, CA125dt, and CA125vel were, respectively, of 0.873, 0.727, and, 0.903. For the SUVmax the optimal cut-off was 2, with an AUC of 0.982. AUC = area under the curve, ROC = receiver-operating characteristics.

The ROC analysis (Fig. 3) demonstrated an optimal cut-off value for CA125 of 23.95 U/mL, achieving a sensitivity of 87.3% and a specificity of 78.6%, with an area under the curve (AUC) of 0.873 (95% confidence interval [CI] 0.777–0.969, *P* < .001) in the prediction of recurrence. About the aim of the kinetic values of CA125 in detecting recurrence we observed, an optimal cut-off for the CA125dt of 3.36 months (sensibility 58.2%, specificity 71.4%) with an AUC of 0.727 (95% CI 0.542–0.913, *P* < .01). On the other hand, the analysis revealed a cut-off for the CA125vel of 4.49 U/mL per mo (sensibility of 63.6%, specificity of 92.9%), with an AUC of 0.903 (95% CI 0.813–0.994, *P* < .001). For the SUVmax an optimal value of 2 was observed, with a sensitivity of 98.2% and a specificity of 92.9%. The AUC for it was 0.982 (95% CI 0.948–1.000, *P* < .001).

The results obtained by multivariate cox regression are shown in Table 3.

**Table 3:**
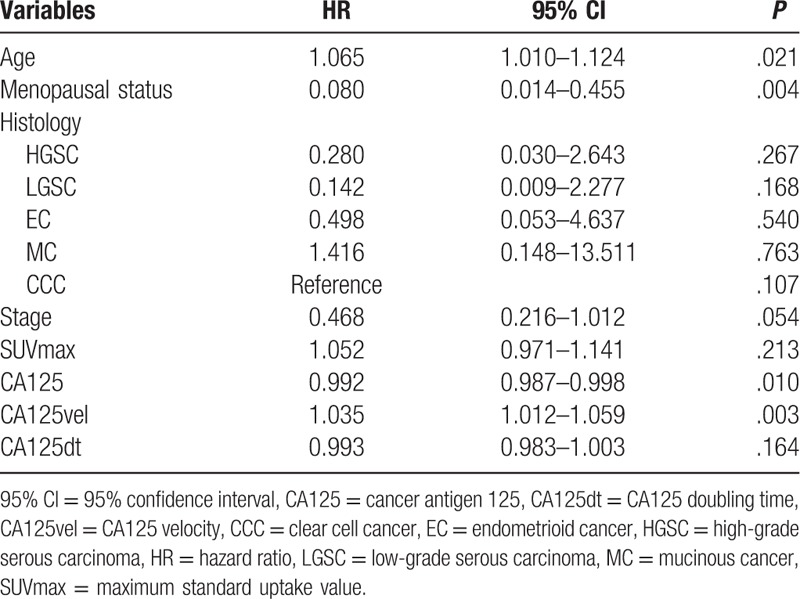
Multivariate cox regression analysis.

## 4 Discussion

An adequate follow-up is necessary in the ovarian cancer, to detect and treat the recurrence as early as possible. If CA125 is used in isolation we could detect the recurrent disease too late, in patients with lower levels than the reference limit, or even we could treat patients with elevated CA125, due to nontumor causes. So, efforts are now directed to improve the accuracy of the measurements to upgrade the precision in the early detection of recurrence. EOC subtypes have been demonstrated that are inherently different diseases. Regarding CA125 serum levels, HGSC and LGSC showed higher concentrations, followed by EC and CCC, while it is less frequent in MC.^[11,12]^ Our work, despite the distribution of histologies resembles that seen all over in ovarian cancer,^[1]^ showed greater CA125 serum levels in HSGC and EC, and lower in LGSC, MC, and CCC, without statistical differences between recurrent and nonrecurrent cases, except in HGSC.

Some authors^[4]^ have recommended that patients must show evidence of CA125 ≥ 2 times the upper limit of the reference range or nadir value on 2 occasions, at least 1 week apart, to be considered in recurrence. In our series, CA125 nadir has not been considered, and that could be a selection bias, but all patients had 2 measurements of CA125 that allowed to calculate kinetic parameters. These parameters, as used in the initial assessment of ovarian cancer,^[13,14]^ may be of utility in assessing recurrence. Thus, beyond the estimation of kinetic values of CA125, helps in the selection of patients referred to perform a [^18^F]FDG-PET/CT to detect early disease. In accordance with our findings, CA125vel is the most useful parameter, due to the high AUC showed in the ROC analysis. This is consistent with that described in the work of Levy et al^[5]^ in which 52 patients were assessed by CA125 elevation above the normal limit. It was evident that patients with a pattern of abrupt rise of CA125 had worse prognosis than patients with a progressive increase.

In our sample, a mean of 24.58 U/mL per mo was observed in the recurrence group, higher than the observed in the nonrecurrence group (0.60 U/mL per mo). Similarly, as previously published in a work with 311 patients, using 25 U/mL per mo as the upper limit of grade of CA125 increase, could allow to achieved a 100% specificity for detecting recurrence.^[15]^ Globally, the difference between CA125vel mean in recurrence and nonrecurrence cases is 23.98 U/mL per mo. This difference increases in case of HGSC, with a mean difference of 31.70 U/mL per mo between recurrent and nonrecurrent cases, while is lower than 10 U/mL per mo in the rest of histologies. Probably, this is due to higher proliferation index that exhibits HGSC in comparison with other histological subtypes.^[1]^ The extension of disease influences the proliferation of the tumor marker, that is the reason why early stages usually exhibit median values for CA125 lower than late stages.^[11,12,16]^ But in our series, there are no statistical differences between both groups, and neither in the kinetic values. This could be due to the extension of the disease in the recurrence (not analyzed in this paper), and not just the initial spread of the disease.

As Prat et al previously published,^[6]^ one of the early signs of recurrence might be a low-level increase in CA125 serum values within the normal range during surveillance for ovarian cancer recurrence. That, as the ROC analysis demonstrated in our sample, a rise of 4.49 U/mL per mo should make us consider the performance of PET/CT to rule out recurrence, without waiting for a large increase of CA125. Even, the multivariate analysis showed that CA125vel is the most influential factor in disease detection. Thus, the calculation of CA125vel, which does not take too long, because it is calculated by a simple formula, should be determined in daily practice to improve recurrence detection, even with very low levels of CA125.

On the other hand, the CA125dt, overall, has not shown differences (*P* = .13) in patients with relapse and nonrecurrence, with a mean difference of 19.41 months between them. The same occurs in histological subtypes, with a nonstatistical difference in any of the subgroup. The longitudinal CA125, which consists in an analysis from diagnosis to completion of first-line chemotherapy, has been proposed to predict recurrence. Because it showed better predictive value than kinetic factors after the treatment, as CA125 half-life, time to reach CA125 nadir and CA125 nadir.^[17]^ Moreover, the literature refers that patients with a doubling time of CA125 shorter than 40 days have a significantly shorter survival than those whose doubling time is >40 days (10.6 months vs. 22.1 months), independently of the time to disease progression.^[18]^ As a justification for our results, without differences in recurrence and nonrecurrence groups, it is important to note, as suggest Xu et al^[13]^ that although the doubling time is the same from 2 to 4 U/mL and from 20 to 40 U/mL, the first offers a little indication of ovarian cancer recurrence, whereas the second one in the same period provides a much stronger indication. Which makes necessary to a better assessment jointly assess CA125 serum level and doubling time, achieve by the CA125vel.

According to our data, while CA125 serum levels have shown a relatively low sensitivity and specificity, [^18^F]FDG-PET/CT has correctly cataloged all cases, regardless of the level of CA125, with a high prevalence of recurrence (79.71%). SUVmax showed statistical differences between recurrent and nonrecurrent cases in distinct histological subtypes. The exception was the mucinous group, in which 1 patient showed an inflammatory activity caused by mesenteric adenitis. That was correctly classified based on the assessment of fusion, metabolic and anatomical images, which are essential for a suitable categorizing of findings, avoiding false positive results due to inflammatory processes. SUVmax is influenced by glucose metabolism, and tumor differentiation, proliferative potential, cell density, as well as mucinous and fibrous components.^[19]^ So mucinous tumors are presupposed to have low [^18^F]FDG uptake, but metabolic rate of tumor varies also depending on intratumoral heterogeneity.^[20]^ But the semiquantitative measurements should not be taken as the only parameter to detect disease, despite the results observed in the ROC analysis, because it is not a “magic point” that can differentiate malignant to benign lesions. Thus, the visual analysis and, therefore, the diagnostic skill of nuclear medicine physicians in the interpretation are essential. Beside the metabolic tumor volume (MTV) and total lesion glycolysis (TLG) are parameters that may help to individually tailor postoperative adjuvant chemotherapy, better than SUVmax, because reflect the metabolic burden and disease extent.^[21]^

**Figure 4:**
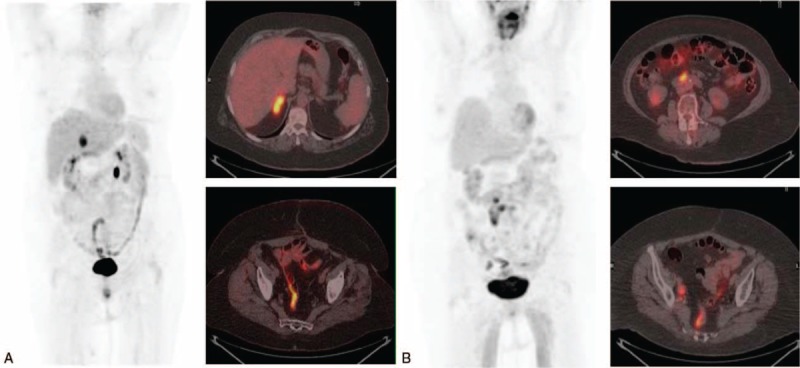
(A) Maximum intensity projection (MIP) and axial fusion positron emission tomography/computed tomography with 2-deoxy-2-[^18^F]fluoro-d-glucose ([^18^F]FDG-PET/CT) in a 68-year-old woman with recurrent ovarian cancer (FIGO stage IIIC, high-grade serous carcinoma, diagnosed 49 months before), with CA125 of 51.6 U/mL, CA125vel of 4.6 U/mL per mo and a doubling time of 7.4. Surgery confirmed the existence of a metastasis on right adrenal gland that showed high [^18^F]FDG uptake (SUVmax 10.6). (B) Woman (70-year-old) with history of mucinous ovarian carcinoma, that showed rising CA125 (62.4 U/mL), with a CA125vel of 5.4 U/mL per mo and CA125dt of 6.73. The [^18^F]FDG-PET/CT, MIP, and axial fusion PET/CT exhibited multiple lymph nodes in retroperitoneal space and both iliac territories. All locations visualized in [^18^F]FDG-PET/CT, with an SUVmax of 7.3, were confirmed histologically. Both cases showed irregular intestinal uptake.

The setting-up of the extent of the disease by PET/CT allowed in 25 cases to perform surgery prior to the consolidation chemotherapy (Fig. 4), while in 30 cases the chemotherapy was administrated. The elevated levels of CA125 were not indicative of tumor recurrence in 3 cases, and the PET/CT prevented treatment administration. This, associated with the fact that chemotherapy based on a raised CA125 concentration alone has not demonstrated improved survival, suggests the need to assess patients with suspected recurrent EOC by PET/CT. That is better than a combination of conventional imaging techniques, to offer site-specific therapy if possible. In the same way, Ebina et al^[22]^ demonstrated that [^18^F]FDG-PET/CT is a useful tool for selecting candidates for cytoreductive surgery. They refer that patients with localized [^18^F]FDG uptake patterns are the best candidates for surgical therapy, and these patients usually have a treatment-free interval of more than 6 months. The information of [^18^F]FDG-PET/CT determines an improvement in restaging accuracy and in the pathway cost. This is because [^18^F]FDG-PET/CT reduces inappropriate surgical procedures with an impact on the patients’ health and on the system expenses by distinguish a single lesion, for which surgery is required, from multiple lesions, for which surgery is not to be performed.^[23]^

The main limitations of our study are derived from its nonrandomized retrospective nature and the small number of patients, especially in the number of non-HGSC. In addition, they were patients with a high prevalence of disease, due to the selection patients from PET/CT database. The inclusion criteria could generate a selection bias, essentially, given the exclusion of early recurrence. However, in our opinion, early recurrence is mostly due to persistent undetectable disease despite treatment. To minimize bias in the assessment of PET/CT nuclear medicine physicians evaluated blindly. Further prospective studies would be necessary to validate the current data, particularly in those patients with early recurrence, considered platinum-resistant.

## 5 Conclusions

[^18^F]FDG-PET/CT has proved to be more accurate than CA125 serum level to detect ovarian cancer recurrence allowing to establish thus earlier the more adequate treatment. CA125vel is a parameter easily calculated, that could better select those patients that may benefit from the performance of a [^18^F]FDG-PET/CT study. A CA125vel of 4.49 U/mL per mo was observed as the best cut-off; so, this variation could be employed to consider a [^18^F]FDG-PET/CT, independently of CA125 serum levels.

The HGSC is the most beneficiary of this calculation, whereas tumor stages present similar performance. Despite SUVmax showed higher levels in recurrent disease, the multivariate analysis showed that CA125 and CA125vel were independent parameters in patients with recurrent EOC.

## Acknowledgments

The authors thank Dr. Muñoz Rodríguez, Translational Research Unit, Hospital General Universitario de Ciudad Real, for his statistical analysis assistance.

## References

[1] Prat J Ovarian carcinomas: five distinct diseases with different origins, genetic alterations, and clinicopathological features. Virchows Arch 2012;460:237–49. 22322322 10.1007/s00428-012-1203-5

[2] Davidson B Tropé C Ovarian cancer: diagnostic, biological and prognostic aspects. Womens Health (Lond Engl) 2014;10:519–33. 25335543 10.2217/whe.14.37

[3] Aust S Pils D Epithelial ovarian cancer—more data, more questions? Wien Med Wochenschr 2014;164:479–86. 25392123 10.1007/s10354-014-0323-8

[4] Rustin G Vergote I Eisenhauer E Definitions for response and progression in ovarian cancer clinical trials incorporating RECIST 1.1 and CA 125 agreed by the Gynecological Cancer Intergroup (GCIG). Int J Gynecol Cancer 2011;21:419–23. 21270624 10.1097/IGC.0b013e3182070f17

[5] Levy T Weiser R Boaz M The significance of the pattern of serum CA125 level ascent to above the normal range in epithelial ovarian, primary peritoneal and tubal carcinoma patients. Gynecol Oncol 2012;129:165–8. 23274778 10.1016/j.ygyno.2012.12.024

[6] Prat A Parera M Adamo B Risk of recurrence during follow-up for optimally treated advanced epithelial ovarian cancer (EOC) with a low-level increase of serum CA-125 levels. Ann Oncol 2009;20:294–7. 18820245 10.1093/annonc/mdn601

[7] Palomar A Nanni C Castellucci P Value of FDG PET/CT in patients with treated ovarian cancer and raised CA125 serum levels. Mol Imaging Biol 2012;14:123–9. 21240639 10.1007/s11307-010-0468-9

[8] Evangelista L Palma M Gregianin M Diagnostic and prognostic evaluation of fluorodeoxyglucose positron emission tomography/computed tomography and its correlation with serum cancer antigen-125 (CA125) in a large cohort of ovarian cancer patients. J Turk Ger Gynecol Assoc 2015;16:137–44. 26401105 10.5152/jtgga.2015.15251 PMC4560469

[9] Fularz M Adamiak P Czepczyński R Utility of PET/CT in the diagnosis of recurrent ovarian cancer depending on CA 125 serum level. Nuklearmedizin 2015;54:158–62. 26076719 10.3413/Nukmed-0709-14-11

[10] Prat J FIGO Committee on Gynecologic Oncology. Staging classification for cancer of the ovary, fallopian tube, and peritoneum. Int J Gynaecol Obstet 2014;124:1–5. 24219974 10.1016/j.ijgo.2013.10.001

[11] Cramer D Vitonis A Welch W Correlates of the preoperative level of CA125 at presentation of ovarian cancer. Gynecol Oncol 2010;119:462–8. 20850174 10.1016/j.ygyno.2010.08.028 PMC2980911

[12] Kristjansdottir B Levan K Partheen K Diagnostic performance of the biomarkers HE4 and CA125 in type I and type II epithelial ovarian cancer. Gynecol Oncol 2013;131:52–8. 23891789 10.1016/j.ygyno.2013.07.094

[13] Xu J Commins J Partridge E Longitudinal evaluation of CA-125 velocity and prediction of ovarian cancer. Gynecol Oncol 2012;125:70–4. 22198243 10.1016/j.ygyno.2011.12.440 PMC3303942

[14] Skates S Menon U MacDonald N Calculation of the risk of ovarian cancer from serial CA-125 values for preclinical detection in postmenopausal women. J Clin Oncol 2003;21:206s–10s. 12743136 10.1200/JCO.2003.02.955

[15] Meier W Baumgartner L Stieber P CA125 based diagnosis and therapy in recurrent ovarian cancer. Anticancer Res 1997;17:3019–20. 9329590

[16] Nakagawa N Koda H Nitta N Reactivity of CA19-9 and CA125 in histological subtypes of epithelial ovarian tumors and ovarian endometriosis. Acta Med Okayama 2015;69:227–35. 26289914 10.18926/AMO/53559

[17] Chang C Chiang A Chen W A joint model based on longitudinal CA125 in ovarian cancer to predict recurrence. Biomark Med 2016;10:53–61. 26565119 10.2217/bmm.15.110

[18] Han L Karavasilis V Hagen T Doubling time of serum CA125 is an independent prognostic factor for survival in patients with ovarian cancer relapsing after first-line chemotherapy. Eur J Cancer 2010;46:1359–64. 20303743 10.1016/j.ejca.2010.02.012

[19] Konishi H Takehara K Kojima A Maximum standardized uptake value of fluorodeoxyglucose positron emission tomography/computed tomography is a prognostic factor in ovarian clear cell adenocarcinoma. Int J Gynecol Cancer 2014;24:1190–4. 24987918 10.1097/IGC.0000000000000180

[20] Lee M Lee H Cheon G Prognostic value of preoperative intratumoral FDG uptake heterogeneity in patients with epithelial ovarian cancer. Eur Radiol 2017;27:16–23. 27121932 10.1007/s00330-016-4368-5

[21] Yamamoto M Tsujikawa T Fujita Y Metabolic tumor burden predicts prognosis of ovarian cancer patients who receive platinum-based adjuvant chemotherapy. Cancer Sci 2016;107:478–85. 26789906 10.1111/cas.12890 PMC4832857

[22] Ebina Y Watari H Kaneuchi M Impact of FDG PET in optimizing patient selection for cytoreductive surgery in recurrent ovarian cancer. Eur J Nucl Med Mol Imaging 2014;41:446–51. 24221243 10.1007/s00259-013-2610-9

[23] Mansuelo M Grimaldi A Mangili G Positron emission tomography/computed tomography introduction in the clinical management of patients with suspected recurrence of ovarian cancer: a cost-effectiveness analysis. Eur J Cancer Care (Engl) 2009;18:612–9. 19549284 10.1111/j.1365-2354.2008.00945.x

